# Effects of a gap between the central and surrounding regions with luminance gradients on the feeling of being dazzled

**DOI:** 10.1177/20416695231176132

**Published:** 2023-05-18

**Authors:** Mitsuhiko Hanada

**Affiliations:** Department of Complex and Intelligent Systems, 73524 Future University Hakodate, Hakodate, Hokkaido, Japan

**Keywords:** luminance gradient, luminance profile, glare, the feeling of being dazzled, gap

## Abstract

The feeling of being dazzled is evoked by images consisting of an achromatic uniform center, surrounded by regions with luminance gradients. As the perceptual distinctness of the central region has been suggested to contribute to the feeling of being dazzled, we examined the effects of a gap between the central and surrounding regions on the feeling of being dazzled. The stimulus comprised a disk with uniform luminance surrounded by an annulus, of which the luminance was decreased from the inner boundary to the periphery. Three luminance profiles (linear, logistic, and inverse-logistic) of the surrounding luminance ramps were used. The distinctness of the disk decreased in the order of logistic, linear, and inverse-logistic profiles. The luminance of the disk, the maximum luminance of the annulus, and the gap size were also varied. When the luminance continuously transitioned from the disk to the annulus, the feeling of being dazzled was stronger for the inverse-logistic annulus luminance profile, compared with the logistic and linear profiles without a gap; however, it was not different for the three profiles with a gap. Further, the feeling of being dazzled increased when a gap was introduced for the logistic and linear profiles, but not for the inverse-logistic profile. These results suggest that the feeling of being dazzled was reduced by the perceptual indistinctness of the central disk for the logistic and linear annulus luminance profiles, while the gap restored the feeling of being dazzled by making the central disk perceptually distinct.

The uniform region surrounded by the gradation pattern, of which the luminance gradually decreases from the central region, appears to be emitting light—or be self-luminous—even if the image is printed on paper and is not, in reality, emitting its own light (Zavagno, 1999). This self-luminous appearance is caused by the surrounding luminance ramp (Zavagno, 1999). Many variations of self-luminosity have been reported (Bressan, 2001; Kennedy, 1976; Zavagno, 1999). Such images, comprising a uniform white region surrounded by luminance gradation, often arise when we take a picture of light sources such as the sun, fire, and illumination lamps; the responses to the light-emitting regions are saturated so that the colors of those saturated regions become uniformly white. Some scatters of light would also occur in the regions surrounding the saturated regions, which creates the halo-like gradation pattern surrounding the central uniform region (Spencer et al., 1995). This phenomenon is referred to as overexposure or whiteout in photography. Overexposure should also occur in retinal photoreceptors when we see a bright light source, and the retinal image should be similar to the overexposed photo. In movies and computer graphics, these types of overexposed images are called glare and have been used to express light sources (Kakimoto et al., 2005; Spencer et al., 1995). This phenomenon of self-luminous appearance due to a luminance ramp is known as the glare effect (Zavagno, 1999; Zavagno & Caputo, 2001, 2005) or glare illusion (Suzuki, Minami, & Nakauchi, 2019; Tamura et al., 2016) in the field of perceptual studies. Such stimuli of a uniform central region surrounded by regions with a luminance ramp that has a glowing appearance do not have a common name. In this study, we refer to these as glare images, as in the study of Hanada (2019).

Glare images appear self-luminous (Facchin et al., 2017; Zavagno, 1999; Zavagno & Caputo, 2001), and the central uniform region of a glare image is perceived to be brighter than that surrounded by a uniform pattern without a luminance ramp (Tamura et al., 2016). Pupils are constricted more by the glare image with surrounding graduation patterns than by control stimuli that do not appear luminous (Kinzuka et al., 2021; Laeng, & Endestad, 2012; Suzuki, Minami, Laeng, & Nakauchi, 2019; Suzuki, Minami, & Nakauchi, 2019; Zavagno et al., 2017); this is consistent with self-luminosity and brightness enhancement by luminance ramps. The surrounding luminance ramps also affect the brightness of adjacent regions, as well as brightness contrast (Agostini & Galmonte, 2002; Zavagno, 2005; Zavagno & Daneyko, 2008). The luminance-reversing (monochrome inversion) pattern of a glare image makes the central black region appear darker (Zavagno, 1999; Zavagno & Daneyko, 2008; but Kobayashi et al., 2021), and evokes pupil dilation (Laeng, Nabil, & Kitaoka, 2022; Zavagno et al., 2017).

Glare images evoke not only greater perceived brightness and pupil constriction but we also feel somewhat dazzled when seeing such images even though we are not actually dazzled because the luminance of such images is not very high. Further, we also feel dazzled when seeing whiteout images of light sources in movies and computer graphics. Viewers of glare images often feel a compulsion to look away from such stimuli or to close their eyes. Here the sensation of wanting to avoid light without the eyes being blinded, nor a sense of pain, is called the feeling of being dazzled (*mabushisa* in Japanese). Evoking feelings of being dazzled seems to be one of the primary reasons for using glare images in movies and computer graphics. Some patients with eye diseases, such as cataracts, complain about being dazzled by strong lights, such as car headlights. The feeling of being dazzled is one component of their symptoms and may be caused by luminance gradients caused by scattering light, as seen through the cloudiness of their eyes.

Hanada (2012, 2015, 2019) studied the feeling of being dazzled using glare images. Hanada's (2012) study examined the effects of luminance profiles in the peripheral luminance ramp on the feeling of being dazzled. Images consisting of a central disk surrounded by an annulus with a luminance gradient were used (Figure 1). Three luminance profiles for the annulus (logistic, linear, and inverse-logistic) were used (Figure 2). When the disk luminance and annulus maximum luminance were the same, the distinctness of the border between the disk and annulus increased in the order of the logistic, linear, and inverse-logistic profiles; this is because the luminance gradient just outside the border becomes larger in that order (Figures 1[a]–[c] and 2). They found that an annulus's luminance profile makes the border between the disk and annulus vague, which reduced the feeling of being dazzled. These findings suggest that indistinctness between the disk and annulus weakens the feeling of being dazzled. In another study, Hanada (2015) varied the colors of the disk and annulus to investigate the influence of color on the feeling of being dazzled. He reported that pink and light blue annuluses evoked a stronger dazzling feeling than gray, green, and yellow annuluses. They also found that the feeling of being dazzled tended to be weaker when the disk and annulus had the same color than when they had different colors. This also suggests that an indistinctness of the central disk from the surrounding annulus weakens the feeling of being dazzled. Hanada (2019) varied not only the luminance, but also the chromaticity of the surrounding annulus, to gradually change the color saturation in the annulus. They found that the saturation gradients that cause a smooth chromaticity transition at the border of the central disk evoked a weaker feeling of being dazzled, compared with the saturation gradient that produced an abrupt chromaticity transition. This report also supports the idea that an indistinctness of the central disk weakens the feeling of being dazzled.

**Figure 1.**
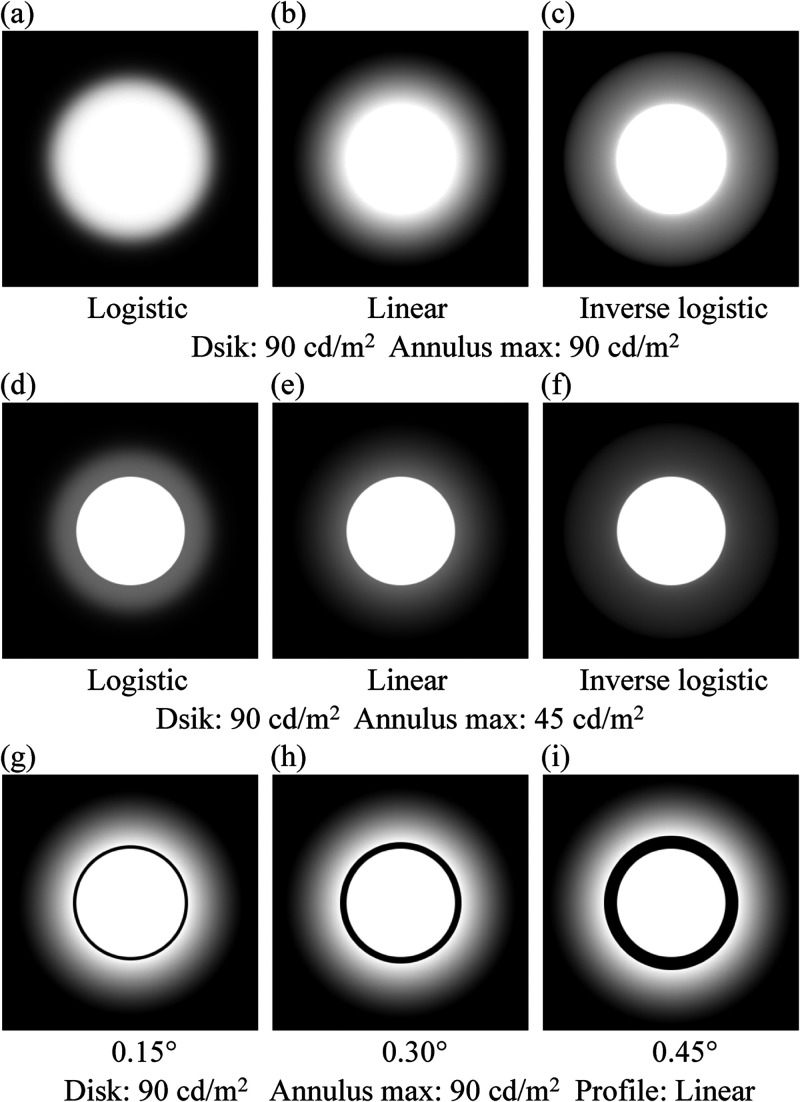
Examples of the images with the logistic [(a) and (d)], linear [(b), (e) and (g)–(i)], and inverse-logistic [(c) and (f)] profiles. The images used for the disk luminance of 90 cd/m^2^ and the annulus luminance of 90 cd/m^2^ are shown in (a)–(c) and (g)–(i). The images used for the disk luminance of 90 cd/m^2^ and the annulus luminance of 45 cd/m^2^ are shown in (d)–(f). The gap size of the images (a)–(f) is 0° (no gap), and that of (g), (i), and (f) is 0.15°, 0.30°, and 0.60°, respectively. Note that the images do not represent designated luminances unless they are displayed on an appropriately calibrated monitor.

**Figure 2.**
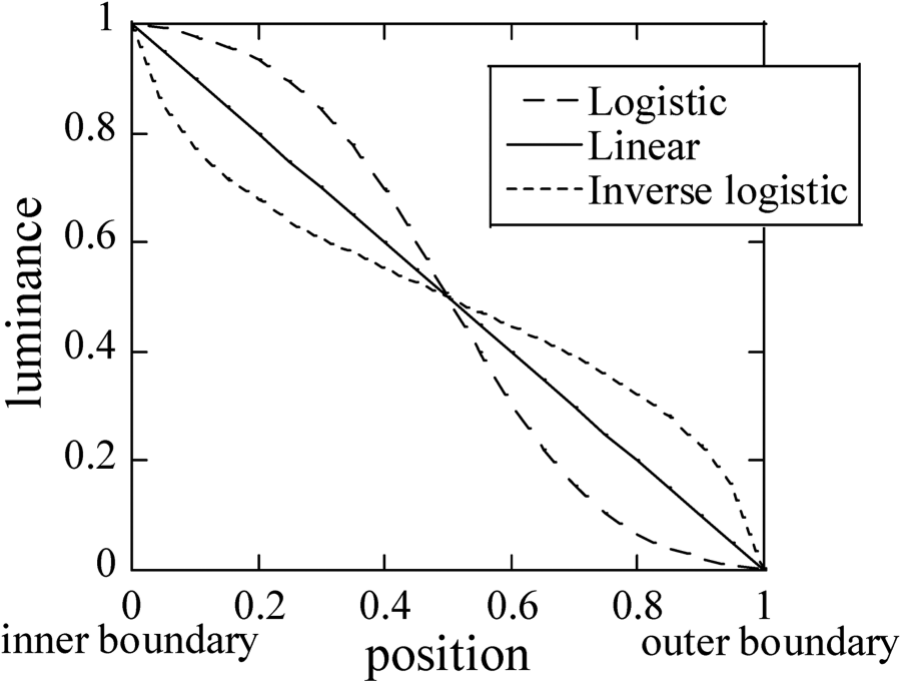
The luminance profiles for the annulus.

Hanada (2012, 2015, 2019) manipulated the distinctness of the central region using luminance profiles and colors. The distinctness can also be manipulated by introducing a gap between the central disk and the surrounding annulus, as shown in Figure 1(g)–(i). This study examined the effects of this gap on the feeling of being dazzled. Hanada (2012) varied the disk luminance, annulus luminance, and the luminance profile of the annulus. Based on this study, we varied the gap size in addition to those stimulus attributes. It should be noted that the disk and annulus luminance were varied because the feeling of being dazzled was too subtle unless they were varied, and the participants paid excessive attention to the details of the stimulus. Moreover, we varied the luminance profile of the annulus to change the central disk's distinctness of the original stimuli without a gap. We hypothesize that introducing the gap between the disk and annulus would increase the feeling of being dazzled when the disk luminance and annulus maximum luminance at the inner border were the same; this is because the gap makes the central disk more perceptually distinct. This gap effect may be the largest for the logistic profile, because the central disk for that profile is indistinct (Figure 1[a]); however, the gap delineates the disk and makes it distinct. The gap effect would be the smallest for the inverse-logistic profile, because the central disk is already distinct for this profile (Figure 1[c]), which means that the increase in distinctness by the gap would be limited. If our experiment supports this hypothesis, it would provide strong evidence that confirms the views of Hanada (2012, 2015, 2019)—that the distinctness/indistinctness of the central uniform region enhances or reduces the feeling of being dazzled.

## Methods

*Apparatus*. We displayed stimuli on the color display of a cathode ray tube (SONY CPD-G420) with a refresh rate of 60 Hz, and were generated by a graphic board (ATI-HD5850) controlled by a computer. This apparatus has the capacity to display 1,024 colors through the R, G, and B channels, each of which has a 10-bit digital-to-analog converter. Participants viewed the display binocularly in a dark room, with their heads supported by a chin rest. The viewing distance was 65 cm, and the display size was 1,024  ×  768 pixels, subtending 30°  ×  23°.

*Participants*. Fifteen Japanese undergraduate or graduate students (22 or 23 years old; *M*  =  22.4, *SD*  =  0.51; 2 women and 13 men) participated in the experiment. All participants were information sciences majors and they were unaware of the purpose of the experiment. Informed consent was obtained from all participants. The study was approved by the ethics committee of Future University Hakodate.

*Stimuli*. The stimulus, without a gap, consisted of a disk surrounded by an annulus. The radius of the disk was 2.6°, and the annulus ranged from 2.6° to 5.1° (Figure 1). The background of the stimulus was black, with its luminance too low to measure, but much less than 0.1 cd/m^2^. The stimulus configuration was the same as those used in Hanada's (2012) study. The disk had a uniform luminance, which was set to 45 or 90 cd/m^2^. The luminance in the annulus decreases from the inner border to the periphery. Three types of luminance profiles were used for the annulus (Figure 2): linear ramp, in which the luminance decreased linearly as a function of the distance from the central disk (Figure 1[b] and [e]); logistic, in which the profile for the ramp was a logistic function (Figure 1[a] and [d]); and inverse-logistic function, in which the profile was an inverse-logistic function (Figure 1[c] and [f]).

The luminance at the outer boundary of the annulus is the same as that of the black background. The maximum luminance at the inner boundary of the annulus (i.e., the maximum luminance of the annulus) was set to 45 or 90 cd/m^2^. For the stimuli with a gap between the disk and the annulus, the annulus spanned from 2.6 + α° to 5.1 + α°, where α indicates the gap size, which was set to 0°, 0.15°, 0.30°, or 0.60°. A gap size of 0° indicates no gap, and a visual angle of 0.15° indicates a length of five pixels. Thus, there were 48 conditions for the stimuli: two disk luminances  ×  two annulus maximum luminances  ×  three luminance profiles for the annulus  ×  four gap sizes. Stimuli without annuli were also used; there were two conditions for stimuli without an annulus: two disk luminances. In total, 50 stimulus conditions were used.

*Procedure*. This procedure was similar to those in the existing studies by Hanada (2012, 2015, 2019). First, participants entered a dark room and waited for 2 min for dark adaptation. Next, all the stimuli used in the experiments were presented to participants sequentially in a random order, for 0.5 s per stimulus, so that they could set an internal standard of the degree of being dazzled. In each trial, the stimulus was presented for 1.0 s. If participants failed to see the stimulus during the presentation, they could see it again by pressing a button, but they were told to avoid using this button. The participants used a gamepad to rate their feeling of being dazzled from 0  =  “not dazzled at all” to 10  =  “extremely dazzled” experienced from the whole stimulus image after observing it. To avoid irrelevant visual stimulation, ratings were obtained using sound. The participants increased or decreased the rating by pressing appropriate buttons on a gamepad, and the recorded voices informed them of the changed rating. The current rating was delivered when the *confirmation* button was pressed. After completing the rating for each stimulus, the participant proceeded to the next trial by pressing the *next* button. Each stimulus was evaluated four times per session. Two hundred trials were conducted per session and the order in which the stimuli were presented was randomized. Each participant took part in two sessions, which means that each participant rated each stimulus image eight times. The participants performed the two sessions successively, with a short break (approximately 5–10 min) between sessions.

## Results

**Figure 3.**
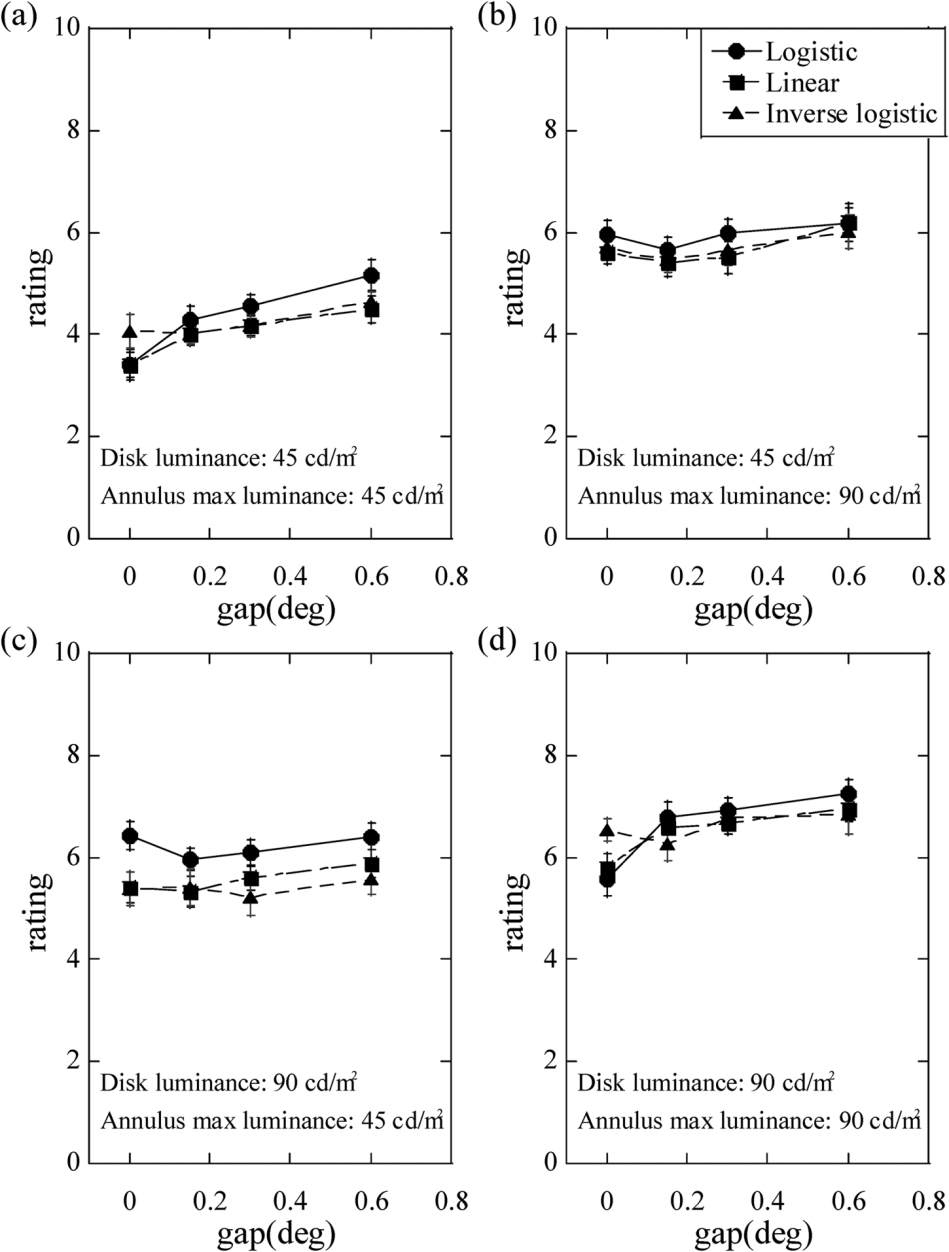
Average ratings of the feeling of being dazzled. The ratings were plotted as a function of the gap size. The error bars represent  ±1 standard error.

Data for the stimuli without the annulus were not included in the statistical analysis, as these conditions did not conform to the experimental design of the stimuli. The overall individual means of the ratings for each stimulus condition are shown in Figure 3. The results of the four-way (disk luminance  ×  annulus maximum luminance  ×  annulus luminance profile  ×  gap size) repeated-measures analysis of variance are shown in Table 1. The main effects of disk luminance, annulus maximum luminance, annulus luminance profile, and gap size were significant. The feeling of being dazzled was stronger for higher disk luminance and annulus maximum luminance, as well as stronger for the logistic annulus luminance profile than for the linear and inverse-logistic profiles. In addition, the ratings were higher for the 0.60° gap than for the other gap sizes. No other significant differences were observed in terms of gap sizes.

**Table 1.** Results of Four-way (Disk Luminance  × Annulus Maximum Luminance  × Annulus Luminance Profile  × Gap) Repeated-Measures ANOVA.

Source	df	MS	*F*	*p*		η_p_^2^
S (participant)	14	17.29				
A (disk luminance)	1	244.20	39.59	<.001	***	0.739
Error[AS]	14	6.17				
B (annulus maximum luminance)	1	268.35	27.33	<.001	***	0.661
Error[BS]	14	9.82				
C (annulus luminance profile)	2	8.62	4.11	.027	*	0.227
Error[CS]	28	2.10				
D (gap size)	3	16.15	10.37	<.001	***	0.425
Error[DS]	42	1.56				
AB	1	23.63	7.97	.014	*	0.363
Error[ABS]	14	2.96				
AC	2	0.97	3.30	.052		0.191
Error[ACS]	28	0.29				
AD	3	0.40	1.33	.279		0.086
Error[ADS]	42	0.30				
BC	2	2.14	5.81	.008	**	0.293
Error[BCS]	28	0.37				
BD	3	0.11	0.41	.750		0.028
Error[BDS]	42	0.27				
CD	6	1.22	4.57	<.001	***	0.246
Error[CDS]	84	0.27				
ABC	2	3.01	9.16	<.001	***	0.396
Error[ABCS]	28	0.33				
ABD	3	7.17	18.12	<.001	***	0.564
Error[ABDS]	42	0.40				
ACD	6	0.06	0.33	.920		0.023
Error[ACDS]	84	0.20				
BCD	6	0.31	1.49	.191		0.096
Error[BCDS]	84	0.21				
ABCD	6	1.94	6.37	<.001	***	0.313
Error[ABCDS]	84	0.30				

*Note*. ANOVA = analysis of variance; MS  =  mean squares. *p < .05, **p < .01, ***p < .001.

**Figure 4.**
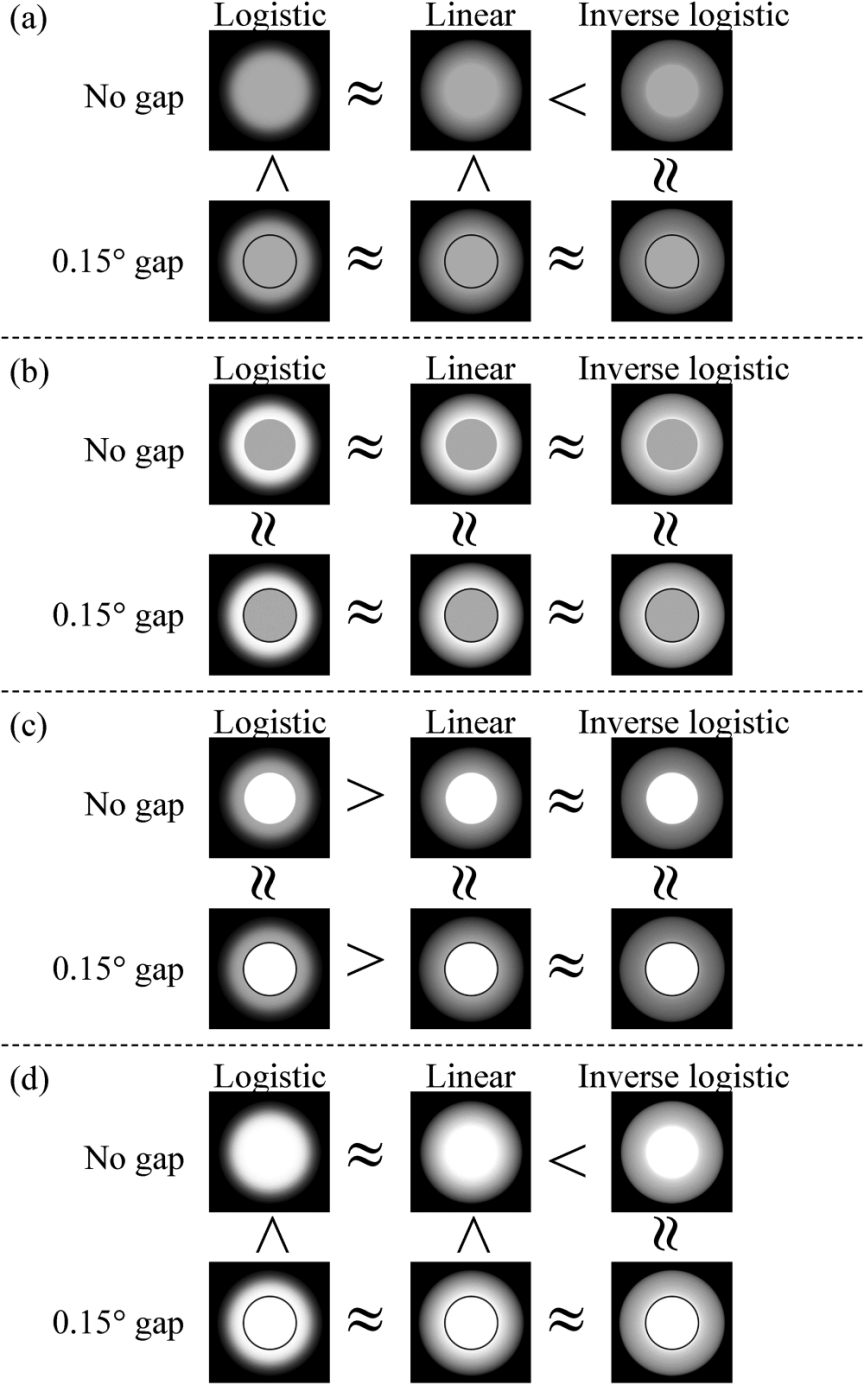
Summary of the significant and nonsignificant differences in the feeling of being dazzled between the annulus luminance profiles for the 0.15° gap and no gap, and those between the 0.15° gap and no gap, for the logistic, linear, and inverse-logistic annulus luminance profiles. The “>” indicates a statistically stronger feeling of being dazzled for the left side, compared with the right side, while the “≈” indicates that the two sides do not differ significantly. (a) Results in the condition of the 45 cd/m^2^ disk luminance and the 45 cd/m^2^ max annulus luminance. (b) Results in the condition of the 45 cd/m^2^ disk luminance and the 90 cd/m^2^ max annulus luminance. (c) Results in the condition of the 90 cd/m^2^ disk luminance and the 45 cd/m^2^ max annulus luminance. (d) Results in the condition of the 90 cd/m^2^ disk luminance and the 90 cd/m^2^ max annulus luminance.

The two-way interactions of disk luminance  ×  annulus maximum luminance, annulus maximum luminance  ×  annulus luminance profile, and annulus luminance profile  ×  gap size were significant, while the three-way interactions of disk luminance  ×  annulus maximum luminance  ×  annulus luminance profile and disk luminance  ×  annulus maximum luminance  ×  gap size were also significant. Most importantly, the four-way interaction of disk luminance  ×  annulus maximum luminance  ×  annulus luminance profile  ×  gap size was significant. Thus, we examined the simple main effect of the annulus luminance profile for each combination of the other stimulus conditions. Table 2 presents the results. Ryan's multiple comparison tests were conducted for significant simple main effects to examine the differences between each pair from the three luminance profiles. The statistically significant differences between the luminance profiles for no gap and the 0.15° gap are summarized in Figure 4, with illustrative images.

**Table 2.** Simple Main Effects of Annulus Luminance Profile for Each Combination of Disk Luminances, Annulus Maximum Luminances, and Gaps for the Stimulus.

Disk Luminance	Annulus Maximum Luminance	Gap Size	df	MS	*F*	*p*		η_p_^2^	Paired Difference
45.0 cd/m^2^	45.0 cd/m^2^	0.0°	2	2.17	5.76	.003	***	0.03	il > lo, il > li
		0.15°	2	0.39	1.02	.360		0.00	
		0.30°	2	0.73	1.93	.146		0.01	
		0.60°	2	1.81	4.80	.009	**	0.02	lo > il, lo > li
45.0 cd/m^2^	90.0 cd/m^2^	0.0°	2	0.47	1.24	.291		0.01	
		0.15°	2	0.25	0.67	.515		0.00	
		0.30°	2	0.85	2.26	.106		0.01	
		0.60°	2	0.14	0.38	.685		0.00	
90.0 cd/m^2^	45.0 cd/m^2^	0.0°	2	5.26	13.96	<.001	***	0.06	lo > il, lo > li
		0.15°	2	1.75	4.64	.010	*	0.02	lo > il, lo > li
		0.30°	2	2.97	7.88	<.001	***	0.03	lo > il, lo > li
		0.60°	2	2.71	7.19	<.001	***	0.03	lo > il, lo > li
90.0 cd/m^2^	90.0 cd/m^2^	0.0°	2	3.85	10.22	<.001	***	0.04	il > lo, il > li
		0.15°	2	1.09	2.88	.057		0.01	
		0.30°	2	0.21	0.57	.567		0.00	
		0.60°	2	0.71	1.89	.152		0.01	
		Error	448	0.38					

*Note*. MS  =  mean squares; lo  =  logistic; li  =  linear; il  =  inverse-logistic. *p < .05, **p < .01, ***p < .001.

The simple main effects of the annulus luminance profile were significant for 45 cd/m^2^ disk luminance and 45 cd/m^2^ annulus maximum luminance, as well as for 90 cd/m^2^ disk luminance and 90 cd/m^2^ annulus maximum luminance without a gap (Figure 3[a] and [d], Table 2). For these stimulus conditions, luminance was continuously changed from the disk to the annulus. Ryan's multiple comparison tests showed that, under these conditions, the feeling of being dazzled was stronger for the inverse-logistic annulus luminance profile than for the logistic and linear profiles (Figure 4[a] and [d]). The simple main effects of the annulus luminance profile were also significant for the 90 cd/m^2^ disk luminance and 45 cd/m^2^ annulus maximum luminance, with any gap size (Figure 3[c] and Table 2). Ryan's multiple comparison tests showed that the feeling of being dazzled was stronger for the logistic annulus luminance profile than for the linear and inverse-logistic profiles under these conditions (Figure 4[c]). In addition, the simple main effects of the annulus luminance profile were significant for 45 cd/m^2^ disk luminance and 45 cd/m^2^ annulus maximum luminance, with a 0.60° gap (Figure 3[b] and Table 2), and the feeling of being dazzled was stronger for the logistic annulus luminance profile than for the other profiles.

We also examined the simple main effect of the gap for each of the other stimulus conditions; the results are shown in Table 3. When the simple main effect was significant, we conducted a Ryan's multiple comparison test. The results were added to Figure 4. When the disk and annulus maximum luminances were the same (45 or 90 cd/m^2^ disk and annulus maximum luminances; Figure 4[a] and [d]), the feeling of being dazzled was stronger for a 0.15° gap than for no gap in the conditions of the logistic and linear annulus luminance profiles; however, the difference between a 0.15° gap and no gap was not significant in the condition of an inverse-logistic profile (Figure 4[a] and [d]). When the disk and annulus maximum luminance were different, the difference between 0.15° and no-gap conditions was not significant for any annulus luminance profile (Figure 4[b] and [c]).

**Table 3.** Simple Main Effects of Gap Size for Each Combination of Disk Luminances, Annulus Maximum Luminances, and Annulus Luminance Profiles for the Stimulus.

Disk Luminance	Annulus Maximum Luminance	Annulus Luminance Profile	df	MS	*F*	*p*		η_p_^2^
45.0 cd/m^2^	45.0 cd/m^2^	Inverse-logistic	3	1.20	3.20	.023	*	0.02
		Linear	3	3.15	8.44	<.001	***	0.05
		Logistic	3	8.01	21.46	<.001	***	0.11
45.0 cd/m^2^	90.0 cd/m^2^	Inverse-logistic	3	0.83	2.21	.086		0.01
		Linear	3	1.88	5.05	.002	**	0.03
		Logistic	3	0.74	1.99	.114		0.01
90.0 cd/m^2^	45.0 cd/m^2^	Inverse-logistic	3	0.35	0.93	.427		0.01
		Linear	3	0.85	2.28	.079		0.01
		Logistic	3	0.80	2.15	.094		0.01
90.0 cd/m^2^	90.0 cd/m^2^	Inverse-logistic	3	1.07	2.85	.037	*	0.02
		Linear	3	3.78	10.13	<.001	***	0.06
		Logistic	3	8.22	22.01	<.001	***	0.12
		Error	504	0.37				

*Note*. MS  =  mean squares. *p < .05, **p < .01, ***p < .001.

## Discussion

This study examined the effects of the gap between a luminous disk and an annulus on participants’ feelings of being dazzled. When the disk luminance and annulus maximum luminance were the same (i.e., the luminance was continuously changed from the disk to the annulus), the feeling of being dazzled was stronger for the inverse-logistic annulus luminance profile than for the logistic and linear profiles without a gap; however, it was not different for the three profiles with a 0.15° gap. Further, the feeling of being dazzled strengthened with the introduction of the gap for the logistic and linear profiles, but not for the inverse-logistic profile. These results suggest that the feeling of being dazzled was reduced by the smooth luminance transition from the disk to the annulus in the logistic and linear profiles and that the gap restored the feeling of being dazzled by separating the central disk from the annulus.

As the previous studies by Hanada (2012, 2015, 2019) showed that the distinctness/indistinctness of the central region induced by the luminance profile and the color difference increases/decreases the feeling of being dazzled, we hypothesized that introducing a gap between the disk and annulus would also increase the feeling of being dazzled, when the disk and annulus maximum luminance were the same. This hypothesis was supported by the logistic and linear annulus luminance profiles (Figures 4[a] and [d]). For the inverse-logistic profile, however, the feeling of being dazzled was not stronger with the 0.15° gap than it was without a gap. For the inverse-logistic profile, the luminance abruptly changes across the border between the disk and annulus, and there appears to be a circular contour between them, even when there is no gap and the central disk is perceptually distinct without a gap. Thus, the gap does not contribute much in terms of distinctness. This should be the reason for the small difference between the 0.15° gap and no-gap conditions for the inverse-logistic profile. This means that the hypothesis was supported as a whole, and the results of this study support the view that the distinctness/indistinctness of the central uniform region evokes a stronger or weaker feeling of being dazzled.

Considering that the logistic annulus luminance profile generally causes a stronger feeling of being dazzled when the disk and annulus maximum luminances are different (Hanada, 2012), it seems that the blurriness of the central disk for the logistic and linear profiles (see Figure 1[a] and [b]) reduces the feeling of being dazzled, while the gap restores the reduced feeling of being dazzled. The disk surrounded by an annulus with a smooth transition from disk to annulus somewhat resembles the shading of a round object. Thus, the visual system may unconsciously process an image not only as a luminous light source, but also as a shaded object; this object processing may inhibit the feeling of being dazzled. However, introducing a gap between the disk and the annulus may stop the processing of object shading and restore the original feeling of being dazzled (Hanada, 2012, 2015, 2019).

The feeling of being dazzled tended to be stronger for the logistic annulus luminance profile, particularly when the luminance of the disk was higher than that of the annulus (Figure 3[c]). A similar result was reported by Hanada et al. (2012). The stronger feeling of being dazzled in the logistic profile may be explained by the stronger luminance around the central disk in the logistic profile than in the linear and inverse-logistic profiles. The feeling of being dazzled may be more related to central luminance than to peripheral luminance.

The gap size affected the feeling of being dazzled; the feeling of being dazzled was stronger for the gap size of 0.60° than for the other gap sizes. When the disk and annulus maximum luminance were the same, the feeling of being dazzled tended to strengthen as the gap size increased, as shown in Figure 3(a) and (d). Considering that the feeling of being dazzled was rated based on the whole image, the effect of the gap size may be explained by the increase in the overall size of the stimulus with the increase in the gap size.

This explanation of the overall stimulus size for the stronger feeling of being dazzled by the gap size of 0.6° raises the possibility that the increase in the overall stimulus size (due to the introduction of the gap size) might contribute to the difference between the 0° and 0.15° gaps for the logistic and linear annulus luminance profiles for the same disk and annulus maximum luminances, as shown in Figure 4(a) and (d). However, it is unlikely, as the gap size is small, and the overall size change with a 0.15° gap is barely perceptible. Moreover, there were no significant differences between the gap sizes of 0° and 0.15° when the disk luminance differed from the annulus maximum luminance (Figure 4[b] and [c]); this suggests that the overall change in stimulus size with the 0.15° gap did not affect the feeling of being dazzled. Furthermore, the overall size variation with different gaps does not explain why the 0.15° gap size did not strengthen the feeling of being dazzled for the inverse-logistic profile, but did for the logistic and linear profiles. The effect of the 0.15° gaps for the logistic and linear annulus luminance profiles shown in Figure 4(a) and (d) can therefore be explained by the indistinctness/distinctness of the disk, as described above, but not by the overall change of the stimulus size with the 0.15° gap.

When the gap size was large, the central disk was surrounded by the dark gap region. Thus, the effect of the gap on the feeling of being dazzled might be explained by enhanced perceived brightness due to the simultaneous brightness contrast evoked by the darkness of the gap region. However, this explanation is unlikely for the following reasons. First, it was reported that the separation has little effect on the simultaneous brightness contrast when the luminance of the inducing region is lower than that of the test region (Leibowitz et al., 1953). Second, brightness contrast cannot explain why the effects of the gap on the feeling of being dazzled were different for the three luminance profiles of the annulus when the disk and annulus maximum luminances were the same (Figure 4[a] and [d]); if the gap effects were caused by simultaneous brightness contrast by the gap, the gap should affect the feeling of being dazzled in the same way for the three luminance profiles. Third, when the disk luminance was 90 cd/m^2^ and the annulus luminance was 45 cd/m^2^, the gap size had little effect on the feeling of being dazzled for all the three luminance profiles of the annulus (Figure 3[c] and Table 2); simultaneous brightness contrast by the gap should affect the feeling of being dazzled in the same way as when the disk and annulus maximum luminances were equal, but the gap effects were different for these cases. Further, simultaneous brightness contrast cannot explain the tendency of stronger feelings of dazzling for the logistic profile, when the central disk is darker than the annulus for the no-gap condition (see also Hanada, 2012). It has been reported that when the center is brighter than the surround, simultaneous brightness contrast is not affected by the surround luminance (Heinemann, 1955) or is generally stronger for the lower surround luminance (Bressan & Actis-Grosso, 2001). As the luminance in the inner half of the annulus was higher for the logistic profile than for the other profiles, the difference in luminance between the disk and the inner part of the annulus was smaller for the logistic profile than for the other profiles. Thus, simultaneous brightness contrast for the logistic profile should have been as strong as, or weaker than, that for the other profiles, and the central disk should have appeared darker for the logistic profile. However, the feeling of dazzling was higher for the logistic profile than for the other profiles.

The effects of the distinctness of the central region might arise, because the participants reported the pronouncedness of the central region confusedly in the experiment of this study and the previous studies of Hanada (2012, 2015, 2019). However, this explanation is unlikely because the participants were told to report the feeling of being dazzled (*mabushisa* in Japanese) as experienced from the whole stimulus and not from a part of it, and were never told anything regarding the pronouncedness. Further, if the participants reported pronouncedness, the ratings should be higher for the disk luminance of 90 cd/m^2^ and the annulus luminance of 45 cd/m^2^ than for the disk luminance of 90 cd/m^2^ and the annulus luminance of 90 cd/m^2^, because luminance was discontinuous between the disk and the annulus in the former condition, which should enhance the pronouncedness. However, the ratings in the former condition tended to be comparable to or slightly lower than those in the latter condition (Figure 3[c] and [d]). The confusion of the perceived brightness of the disk with the feeling of being dazzled cannot also explain the results. The ratings were fairly high for the disk luminance of 45 cd/m^2^ and annulus maximum luminance of 90 cd/m^2^ (Figure 3[c]), and comparable with those for the 90 cd/m^2^ disk luminance and 90 cd/m^2^ annulus maximum luminance. However, if participants rated the brightness of the disk, the ratings should be much lower and would be comparable with those for the disk luminance of 45 cd/m^2^ and annulus maximum luminance of 45 cd/m^2^.

Informal observations show that, while the disk appears luminous when the disk and annulus maximum luminances are 90 cd/m^2^ (Figure 1[a]–[c]), the introduction of a gap between the disk and annulus reduces the self-luminous (light-emitting) appearance. If the feeling of being dazzled is caused by the perceived luminosity of the glare image, the feeling of being dazzled would be stronger without a gap than with it. However, the feeling of dazzling was stronger with a gap than without it for the logistic and linear annulus luminance profiles and showed no difference between the no-gap and the 0.15° gap for the inverse-logistic profile. This indicates that the gap strengthened the feeling of being dazzled in some conditions, despite the disappearance of the self-luminous appearance. This result suggests that the feeling of being dazzled is not entirely linked with perceived self-luminosity.

Informal observation also shows that when the disk and annulus maximum luminances are 90 cd/m^2^ (Figure 1[b] and [c]), a bright white and strongly luminous circular band appears in the outermost region of the disk, for the linear and inverse-logistic profiles of the annulus ramp. A similar light gray band appears when the disk and annulus maximum luminances are 45 cd/m^2^ for the linear and inverse-logistic profiles. This appears to be a type of Mach band, an optical illusion caused by a luminance gradient named after the physicist Ernst Mach (Lotto et al., 1999). It was reported that Mach bands weaken or disappear as the sharpness of the edges between the region of the luminance ramp and the adjacent uniform bright and dark regions decreases (Wallis & Georgeson, 2012), which is consistent with the absence of Mach band for the logistic luminance profile of the annulus, and the presence for the linear and inverse-logic luminance profiles. Mach bands also disappear with an adjacent bar, regardless of the contrast, contrast polarity, and width of the bar (Ratliff et al., 1983). The bright ring pattern actually disappears when a gap is introduced between the disk and annulus for the linear and inverse-logistic luminance profiles. However, the feeling of being dazzled is stronger with a gap for those luminance profiles (Figure 4[d]). This indicates that the feeling of being dazzled is not directly related to the illusion of Mach bands.

As the Mach bands are the optical illusion of brightness, this also suggests that the change in perceived brightness does not directly lead to a change in the feeling of being dazzled. This dissociation of the feeling of being dazzled from perceived brightness may be explained by different contributions of melanopsin containing intrinsically photosensitive retinal ganglion cells to brightness perception and to the feeling of being dazzled. The melanopsin signals affect visual discomfort (Spitschan et al., 2017) and the feeling of being dazzled (*mabushisa*; Higashi et al., 2022) as well as pupil responses (Gamlin et al., 2007), and photophobia and reflexive eye closure in migraine (Kaiser et al., 2021; McAdams et al., 2020). Although the melanopsin signals also affect brightness perception (Brown et al., 2012; Yamakawa et al., 2019), they seem to contribute more to visual discomfort and nonvisual responses such as eye blink and pupil responses than to bright perception. Since the feeling of being dazzled is a component of visual discomfort, it may be affected more by melanopsin signals than brightness perception. Further, the effects of the melanopsin signal on brightness perception may be mediated by visual discomfort or the feeling of being dazzled. Suzuki, Minami, Laeng, & Nakauchi (2019) suggested that the past experience of being dazzled by the Sun may affect brightness perception. Thus, the melanopsin signals may affect the discomfort, which may then weakly modulate brightness perception.

The effects of the annulus luminance profile on the feeling of being dazzled were examined by Hanada (2012), and stimuli without a gap used in this study were also included in that study. Although the overall results were similar between the two studies, there were some discrepancies. When the disk luminance and annulus maximum luminance were the same, the feeling of being dazzled was stronger for the inverse-logistic profile than for the other profiles in the current study, while there were no differences between the profiles in Hanada's (2012) study. Although this difference may be caused by random variations in the results, it is more likely that the feeling of being dazzled was affected by other stimuli; the results would depend somewhat on the context or how to embed the stimulus conditions in question into other stimulus conditions. Although there were slight discrepancies between the two studies, the overall results were similar, and the differences were small. In addition, they did not affect the overall interpretations of the results, especially the conclusion that the feeling of being dazzled was weaker for the indistinct disk than for the distinct disk.

This study has some limitations. There were only 15 participants, which is a fairly small sample. Furthermore, all the participants were university students, and it is unknown whether the findings can be generalized to other populations, such as older adults. In addition, there are some discrepancies between the study of Hanada (2012) and this study regarding the effect of the annulus luminance profile on the feeling of being dazzled, as described above; however, the reasons for these discrepancies are not clear. Further studies are required to address this issue. Despite these limitations, this study provides strong evidence that the distinctness/indistinctness of the central uniform region is an important factor in the feeling of being dazzled. The visual system that evokes the feeling of being dazzled should include mechanisms that explain the findings of this study.
